# The effectiveness of kinesiology taping on dysphagia in brain tumor survivors after neurosurgery: study protocol for a pilot randomized controlled trial

**DOI:** 10.3389/fmed.2025.1571591

**Published:** 2025-10-31

**Authors:** Júlio Belo Fernandes, Leonor Monteiro, Abílio Costa, Ana Sofia Gonçalves, John Dean, Carlos Família, Josefa Domingos, Catarina Godinho

**Affiliations:** ^1^Egas Moniz Center for Interdisciplinary Research (CiiEM), Egas Moniz School of Health & Science, Almada, Portugal; ^2^Nurs* Lab, Almada, Portugal; ^3^Unidade Local de Saúde de Almada-Seixal, Almada, Portugal; ^4^Triad Health, Aurora, CO, United States; ^5^Molecular Pathology and Forensic Biochemistry Laboratory (MPFBL), Costa da Caparica, Portugal

**Keywords:** stroke, deglutition disorders, dysphagia, swallowing therapy, kinesiology taping, rehabilitation

## Abstract

Dysphagia is a common complication in brain tumor survivors, either as a direct symptom of the tumor or a result of neurosurgery. With improved survival rates, the need for effective rehabilitation strategies is more crucial than ever. Kinesiology taping has shown promise in dysphagia rehabilitation for stroke patients, but its potential in brain tumor survivors is largely unstudied and represents a significant research opportunity. This pilot study aims to assess feasibility and gather preliminary data on the impact of kinesiology taping, in addition to standard dysphagia care, on improving swallowing function in brain tumor survivors post-neurosurgery. This 1:1 parallel-group randomized controlled trial will recruit 62 brain tumor survivors with oropharyngeal dysphagia from a Neurosurgery Inpatient Unit in Portugal. Participants will be randomized into two groups: the control group, receiving standard dysphagia care, and the experimental group, receiving kinesiology taping applied to the suprahyoid muscles in addition to standard care. The intervention will last 15 sessions over 4 weeks. Primary outcomes will be assessed using the FEES Penetration-Aspiration Scale, Gugging Swallowing Test (GUSS), Dysphagia Handicap Index (DHI), and Functional Oral Intake Scale (FOIS). This study will evaluate the efficacy of kinesiology taping as a supplementary therapy for dysphagia rehabilitation in brain tumor survivors. The potential improvements in clinical care and patient outcomes are significant, reinforcing the importance of this research in enhancing the quality of life for these survivors.

## 1 Introduction

Kinesiology taping is a widely used therapeutic technique in rehabilitation, frequently applied to support musculoskeletal function, reduce pain, and enhance motor performance (1–3). Over the past decades, its clinical popularity has expanded across a wide range of conditions, from sports injuries to neurological disorders (2, 4).

Recently, interest has grown in exploring the potential of kinesiology taping beyond traditional musculoskeletal contexts, including its application in addressing complex neuromotor functions such as swallowing (5, 6).

Dysphagia is a frequent and often debilitating complication in individuals who have undergone neurosurgery for brain tumors. It may arise as a direct consequence of the tumor’s location and mass effect, or emerge postoperatively due to surgical trauma and neurological impairment (7, 8). Studies indicate that up to 47% of patients with brain tumors may require nasogastric tube feeding in the immediate postoperative period due to dysphagia (9). The incidence of dysphagia after neurosurgery may vary depending on the type of procedure performed, the extent of the surgery, the location of the tumor or lesion, and the patient’s overall health status. Nonetheless, studies suggest dysphagia is a relatively common complication after neurosurgery (10, 11). This condition not only compromises nutritional intake and increases the risk of aspiration pneumonia but also negatively impacts the overall quality of life and recovery trajectory in brain tumor survivors.

With significant advancements in surgery, chemotherapy, and radiotherapy, patients diagnosed with brain tumors now experience a higher survival rate (12–14). Consequently, it has become essential to focus on addressing any lingering neurological impairment and enhancing survivors’ quality of life (15, 16). In this sense, rehabilitation therapy has gained more attention as a valuable approach after brain tumor removal or reduction (15, 17). Furthermore, since stroke and brain tumors often result in comparable symptoms, such as cognitive, motor, and sensory dysfunctions, it is unsurprising that stroke and brain tumor survivors who performed similar rehabilitation programs achieve comparable outcomes (7, 18).

The use of kinesiology taping has generated positive results in a handful of early studies in rehabilitating dysphagia in stroke patients (5, 6, 19). In the pharyngeal phase of swallowing, the suprahyoid muscles are responsible for elevating and stabilizing the hyoid bone and larynx, contributing to proper swallowing mechanisms, including airway protection and opening of the upper esophageal sphincter. If the suprahyoid muscles are weak or damaged, it can result in dysphagia (20, 21). Therefore, strengthening the suprahyoid muscles can be essential to dysphagia rehabilitation to improve swallowing function and reduce the risk of complications (22). In addition, previous studies show that using kinesiology taping to strengthen the suprahyoid muscles can be an effective dysphagia rehabilitation intervention in stroke survivors (5, 6, 19). Thus, given these findings, we present the protocol of a combined intervention using dysphagia training and kinesiology tape application on brain tumor survivors. Therefore, this randomized controlled trial aims to assess the effects of kinesiology tape application in addition to usual care on dysphagia in brain tumor survivors after neurosurgery.

## 2 Methods

### 2.1 Design

This clinical trial is designed as a randomized controlled clinical trial, utilizing a 1:1 allocation ratio. All stages follow the Standard Protocol Items: Recommendations for Interventional Trials - SPIRIT reporting guidelines (23). The trial protocol has been registered with the Australian New Zealand Clinical Trials Registry (registration number: ACTRN12625001071415p).

### 2.2 Study setting

The study will be conducted in collaboration with a Hospital Centre in Portugal. The program will be delivered in the Neurosurgery Inpatient Unit.

### 2.3 Participants

Participants will be recruited from the Neurosurgery Inpatient Unit by the senior nurse manager, specialist in rehabilitation, and the neurologist/neurosurgeon.

Patients with dysphagia after brain tumor surgery will be included in this study if (a) diagnosis of oropharyngeal dysphagia, confirmed by flexible endoscopic evaluation of swallowing (FEES), (b) a Mini-Mental Status Examination (MMSE) score of 22 or higher, (c) ability to swallow voluntarily, (d) ability to swallow against resistance of tape, and (e) able to communicate with the investigator, to understand and comply with the study procedures.

Participants will be excluded if they have (a) dysphagia due to drug toxicity, (b) difficulty with or the inability to trigger a volitional swallow, (c) skin disorders, including allergies associated with taping attachment, (d) developed pneumonia or unstable medical conditions during the study, (e) presence of tracheotomy, (f) known history of progressive neurological disorder (for example, Parkinson’s disease, multiple sclerosis), (g) previously received swallowing therapy, (h) under 18 years of age, and (i) severe cognitive difficulties or significant active psychiatric disorders.

Any subjects who develop pneumonia or other unstable medical conditions at any point will be removed from the study and remanded to the best medical care.

### 2.4 Sample size calculation

A repeated measures ANOVA with within-between interactions was chosen to evaluate the impact of the treatment on each group relative to their baseline. To achieve 80% power, with an alpha of 0.05, a medium effect size of 0.25, two groups, two measurements per group, a correlation of 0.5 among repeated measures, and a non-sphericity correction of 1, we used GPower v.3.1 (24) to calculate the required sample size, which resulted in 34 subjects per group. Considering a 15% dropout rate, the final sample will consist of 80 individuals (*n* = 40 per group) who meet the inclusion criteria.

### 2.5 Randomization and blinding

**FIGURE 1:**
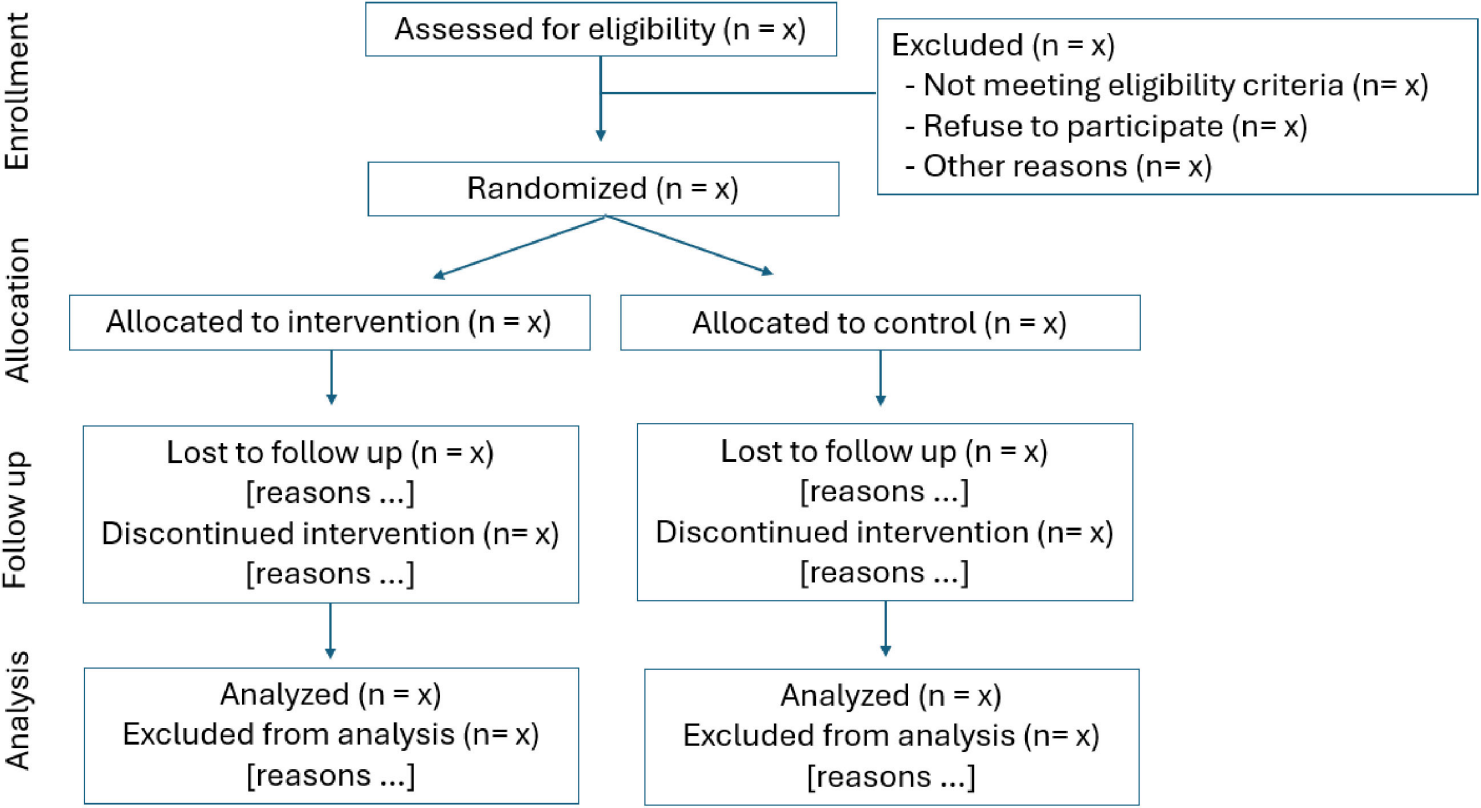
Flowchart describing the search strategy.

Following baseline assessments, participants will be split into two groups: an experimental group and a control group. The randomization plan, including allocation groups and follow-up measurements (as shown in Figure 1), will be generated using online software that employs a random block sizes method. This randomization method guarantees an allocation concealment format, meaning that the person responsible for the task will be unaware of the next group allocation. Due to the protocol differences, it is not feasible to blind the participants or treating clinicians to the condition. Nonetheless, the research team member performing data collection and analysis will be blinded to the group allocation, whether control or experimental.

### 2.6 Intervention

The control group will receive standard dysphagia care, while the intervention group will receive both standard and kinesiology tape applications.

Routine dysphagia care consists of oral exercises (e.g., repeated dry swallowing five consecutive times, followed by a 10-s rest period. This sequence will be repeated a total of 10 times), compensatory techniques (e.g., chin tuck, head rotation, and head tilt), swallowing therapeutic maneuvers (e.g., supraglottic swallowing, super-supraglottic swallow, effortful swallowing, and the Mendelsohn maneuver), and other exercises used to improve swallowing physiology (e.g., the Shaker exercise and Masako maneuver).

This routine will be implemented 4 days a week, with one daily session. Sessions will last approximately 30 min, resulting in 15 sessions.

Sessions will be led by a team of two senior nurses specialized in rehabilitation, experts in dysphagia care, and trained in kinesiology taping application, with more than 10 years of clinical experience.

**FIGURE 2:**
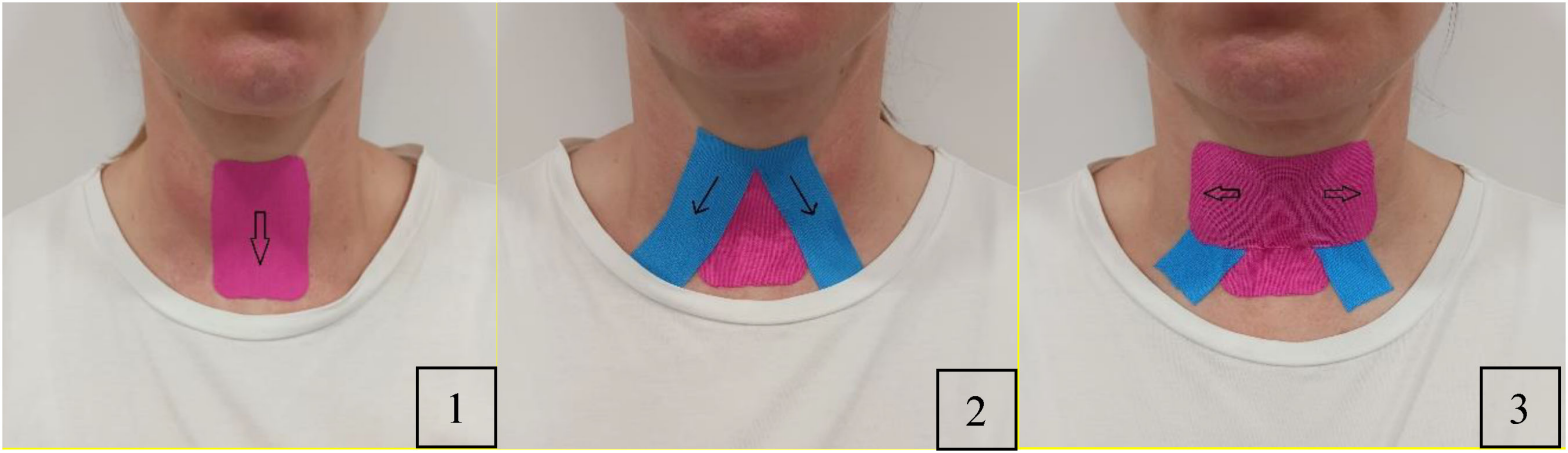
Application of kinesiology taping.

A 5 cm wide, 0.5 mm thick kinesiology tape will be applied as follows: Participants will sit upright in a chair with their head and neck in a neutral position. The anterior neck will be cleaned with an alcohol swab for optimal tape adhesion. The application will follow the method outlined by Park et al. (25) (Figure 2).

Team members will customize an I-shaped tape and a reverse V-shaped tape. The hyoid bone and thyroid cartilage will be marked with a pen. First, an I-shaped tape will be applied by pulling it downward to the level of the thyroid notch to wrap around the thyroid cartilage and attach it to the sternum. Second, the team members will attach a reverse V-shaped tape from the hyoid bone to the superior medial surface of the clavicle on both sides. Finally, they will cover the hyolaryngeal complex horizontally to restrict its movement throughout swallowing, ensuring the tape is securely attached.

Tapes will be replaced daily with an application at approximately 70%–80% tension.

### 2.7 Concomitant treatments and medication

Participants can continue their routine medications for underlying diseases such as hypertension and diabetes. However, the use of additional benzodiazepines, antipsychotics, and anti-epileptics that may impact their swallowing function is permitted only if deemed necessary. If a participant takes medication that could affect their swallowing ability, it will be documented in the case report form.

### 2.8 Data collection

We will use the participants’ clinical records to collect the following characteristics: Participants’ sex, age, educational level, occupation, brain tumor history, and comorbidities.

A blinded researcher will assess primary study outcomes at baseline *T0* and after intervention *T1* (until 24 h after the final session).

The following domains will assess the primary outcomes of this study:

1. The Fiberoptic Endoscopic Evaluation of Swallowing (FEES) will be used alongside the Penetration-Aspiration Scale (PAS). Participants will be asked to swallow solid, semi-liquid, and liquid food. The assessment will be stopped if aspiration occurs, defined as the entry of food into the airway below the true vocal cords (26). The PAS is an 8-point scale that evaluates the severity of food or liquid entering the airway during swallowing, based on FEES observations. It assesses how far the material penetrates and whether it is cleared. Scores range from no airway entry to material entering below the vocal cords, not being cleared, and no protective response (silent aspiration).
2. Gugging Swallowing Test (GUSS). The GUSS is a clinical tool used to assess the safety of swallowing in patients and identify the risk of aspiration. The test evaluates the patient’s ability to swallow different food and liquid consistencies, beginning with thickened fluids and gradually progressing to thin liquids. The GUSS is divided into two main parts: the indirect swallowing test, which assesses the patient’s ability to swallow saliva and checks for symptoms of dysphagia, such as coughing or drooling, and the direct swallowing test, which observes for signs of aspiration or choking as the patient is given substances of increasing difficulty (semisolid, liquid, and solid).

The maximum score indicates safe swallowing without aspiration risk, while lower scores suggest higher aspiration risk.

1. Dysphagia Handicap Index (DHI). The DHI is a self-reported questionnaire designed to assess the impact of dysphagia swallowing on an individual’s daily life. It evaluates three key dimensions: physical, functional, and emotional aspects of living with dysphagia. The DHI consists of 25 items, scored using a Likert scale, with higher scores indicating a more significant perceived handicap.
2. Functional Oral Intake Scale (FOIS). The FOIS is a clinical tool used to evaluate a patient’s ability to consume oral foods and liquids. It provides a standardized measure of functional oral intake, particularly in individuals with dysphagia. The scale consists of 7 levels, ranging from complete reliance on non-oral feeding to the ability to eat and drink without restrictions.

The secondary outcome of this study will be evaluated at T1 by the following key domains:

(1)    Recruitment (number of patients that express interest in participating);
(2)    Enrollment (number of patients that participate in the program);
(3)    Retention (number of sessions and percentage of enrolled participants who completed final program assessments);
(4)    Satisfaction (assessed with an exit survey) will be recorded using binary response questions (yes/no) and a 5-point Likert-type scale;
(5)    Adverse events that happen throughout the intervention (will be documented in the case report form).

### 2.9 Data analysis

Descriptive statistics measures of mean, standard deviation, median, minimum, maximum, and range will be used to characterize the sample in terms of sociodemographic characteristics and variables of interest for each group in the study. In addition, boxplots will also be created to show these variables in a graphical format. Subsequently, a repeated measures analysis of variance (ANOVA) will be used to identify the differences between the two groups and evaluate which exhibits better outcomes. The assumptions of the ANOVA will be verified through the Mauchly’s test of sphericity, the Levene test for homogeneity of variances and visual observation of the Q-Q plot.

### 2.10 Ethics and dissemination

To ensure ethical compliance, the research will adhere to the Helsinki Declaration (2013 revision) and undergo evaluation by the institution’s Ethics Committee. Before any procedures, participants will receive an informed consent form outlining comprehensive details regarding the study’s objectives, procedures, voluntary participation, and potential risks. Participants will be required to sign the form indicating their agreement.

Researchers will emphasize that participants are free to withdraw their consent at any time without prejudice to future medical care. Participants declining to provide or retracting written informed consent will be excluded or discontinued from the research, but this decision will have no bearing on their ongoing care. During the screening visit, participants will receive a comprehensive explanation of the study’s objectives and the expected level of compliance. Any queries or concerns will be addressed. If participants voluntarily consent to participate, they will be asked to sign two copies of the informed consent form. One copy will be provided to the participant, while the leading researcher will retain the other. Data collection instruments will be encoded using a combination of letters and numbers to ensure participants’ anonymity. The decoder grid will only be accessible to the project manager. All paper and electronic data will be stored in a locked file in a cabinet at Egas Moniz - Cooperativa de Ensino Superior, C.R.L, for a 5-year retention period, after which they will be destroyed.

## 3 Discussion

The results of this randomized controlled trial could have important implications for the management of dysphagia in brain tumor survivors after neurosurgery. Dysphagia is a significant complication following neurosurgical interventions, particularly in brain tumor patients, as it can severely affect nutritional intake, hydration, and overall quality of life (27). If proven effective, Kinesiology taping could offer a novel, non-invasive, and easily applicable therapeutic option that complements existing treatments and enhances rehabilitation outcomes.

Evidence from previous studies suggests that kinesiology taping has beneficial effects on stroke survivors with dysphagia (6, 19), likely due to its impact on neuromuscular function. By providing external support and facilitating proper muscle alignment, kinesiology taping may enhance muscle coordination and proprioception in the muscles involved in swallowing (6, 19). For brain tumor survivors, who often experience post-surgical muscle weakness and impaired neuromuscular control (28), applying kinesiology tape could help improve the biomechanics of swallowing, thereby reducing the risk of aspiration and enhancing safety and efficiency in swallowing.

Additionally, this study addresses a significant gap in literature. Although dysphagia rehabilitation has been studied in stroke patients, few studies have focused on brain tumor survivors despite the high prevalence of swallowing difficulties in this population (8, 29). Brain tumors, especially those in areas responsible for motor control or sensation, can lead to significant neuromuscular impairments (28). Post-surgical dysphagia can further complicate recovery, as these patients often require additional treatments such as radiotherapy or chemotherapy, which can exacerbate swallowing difficulties (30, 31). This trial will be the first to examine kinesiology taping as a rehabilitative intervention for this specific patient population, potentially laying the groundwork for future research.

If kinesiology taping is effective, it could have far-reaching implications for clinical practice. Rehabilitation professionals could incorporate this technique into standard dysphagia care, potentially reducing the need for more invasive interventions. Moreover, since kinesiology taping is relatively inexpensive and non-invasive, it could be particularly beneficial in settings with limited access to specialized rehabilitation services. The ease of application and potential for home use also means that patients could continue their rehabilitation outside the clinical setting, empowering them to take a more active role in their recovery and improving long-term outcomes.

While the results will remain general due to the pilot nature of the study, it is essential to emphasize the importance of developing standardized protocols for managing neurological swallowing disorders (32, 33). Such protocols are fundamental to ensuring consistency and quality of care across different clinical settings. They support the appropriate selection and timing of assessment tools and guide the implementation of evidence-based therapeutic strategies. Moreover, standardized protocols may foster effective interdisciplinary collaboration. This comprehensive approach can enhance diagnostic accuracy, streamline intervention delivery, and improve patient outcomes. Establishing and validating these protocols in populations such as brain tumor survivors is a critical step toward reducing variability in care and optimizing the rehabilitation trajectory for individuals with complex neurological impairments.

### 3.1 Strengths and limitations

Although this randomized controlled trial is the pioneer in exploring the effectiveness of kinesiology taping for treating dysphagia in survivors of brain tumors after undergoing neurosurgical intervention, we have built the protocol based on previous work examining the effectiveness of kinesiology taping on stroke survivors, thus expecting to achieve positive outcomes. In addition, the chosen outcome measures represent those most practically implemented in the clinical settings.

To ensure this randomized controlled trial was designed following trial standards, we used the SPIRIT statement, which is widely endorsed as an international standard for trial protocols, to enhance the quality, transparency, and completeness of trial protocols, which can help to improve the overall reliability and validity of this clinical trial results. Nonetheless, it should be noted that this trial has limitations. First, blinding is essential to minimize bias that can affect study outcomes. In this trial, it is not feasible to blind participants or team members who will implement the intervention. However, to minimize data collection and analysis bias, the research team members responsible for these tasks will be blinded to the treatment allocation, whether the participant received the intervention or the placebo/control. Second, there is no uniform standard care treatment for people with dysphagia in Portugal. For this reason, team members will perform the usual treatment in the Neurosurgery Inpatient Unit, bearing in mind that the time and periodicity of the intervention applied in this trial are possibly more prolonged than the time of rehabilitation care practiced in other similar units. Third, we expect some participants to not complete the intervention due to being transferred to other units or facilities (e.g., rehabilitation units). To minimize the impact of this occurrence on the research results, we assume a dropout rate of 15% when calculating the study’s sample size.

## 4 Conclusion

Kinesiology taping has the potential to be an effective treatment option in brain tumor survivors after undergoing neurosurgery. The study of the effectiveness of new therapeutic approaches in dysphagia care is essential to improve patient outcomes and quality of life. Studying these new approaches can help to identify potential risks and side effects associated with these approaches and determine which approaches are most effective for different populations. Healthcare professionals and researchers should continue exploring new approaches to dysphagia care and study their effectiveness to ensure patients receive the best care.
